# Steroid receptor–DNA interactions: toward a quantitative connection between energetics and transcriptional regulation

**DOI:** 10.1093/nar/gkt859

**Published:** 2013-09-21

**Authors:** David L. Bain, Keith D. Connaghan, Nasib K. Maluf, Qin Yang, Michael T. Miura, Rolando W. De Angelis, Gregory D. Degala, James R. Lambert

**Affiliations:** ^1^Department of Pharmaceutical Sciences, University of Colorado Anschutz Medical Campus, Aurora, CO 80045, USA and ^2^Department of Pathology, University of Colorado Anschutz Medical Campus, Aurora, CO 80045, USA

## Abstract

Steroid receptors comprise an evolutionarily conserved family of transcription factors. Although the qualitative aspects by which individual receptors regulate transcription are well understood, a quantitative perspective is less clear. This is primarily because receptor function is considerably more complex than that of classical regulatory factors such as phage or bacterial repressors. Here we discuss recent advances in placing receptor-specific transcriptional regulation on a more quantitative footing, specifically focusing on the role of macromolecular interaction energetics. We first highlight limitations and challenges associated with traditional approaches for assessing the role of energetics (more specifically, binding affinity) with functional outcomes such as transcriptional activation. We next demonstrate how rigorous *in vitro* measurements and straightforward interaction models quantitatively relate energetics to transcriptional activity within the cell, and follow by discussing why such an approach is unexpectedly effective in explaining complex functional behavior. Finally, we examine the implications of these findings for considering the unique gene regulatory properties of the individual receptors.

## INTRODUCTION

**Figure 1.**
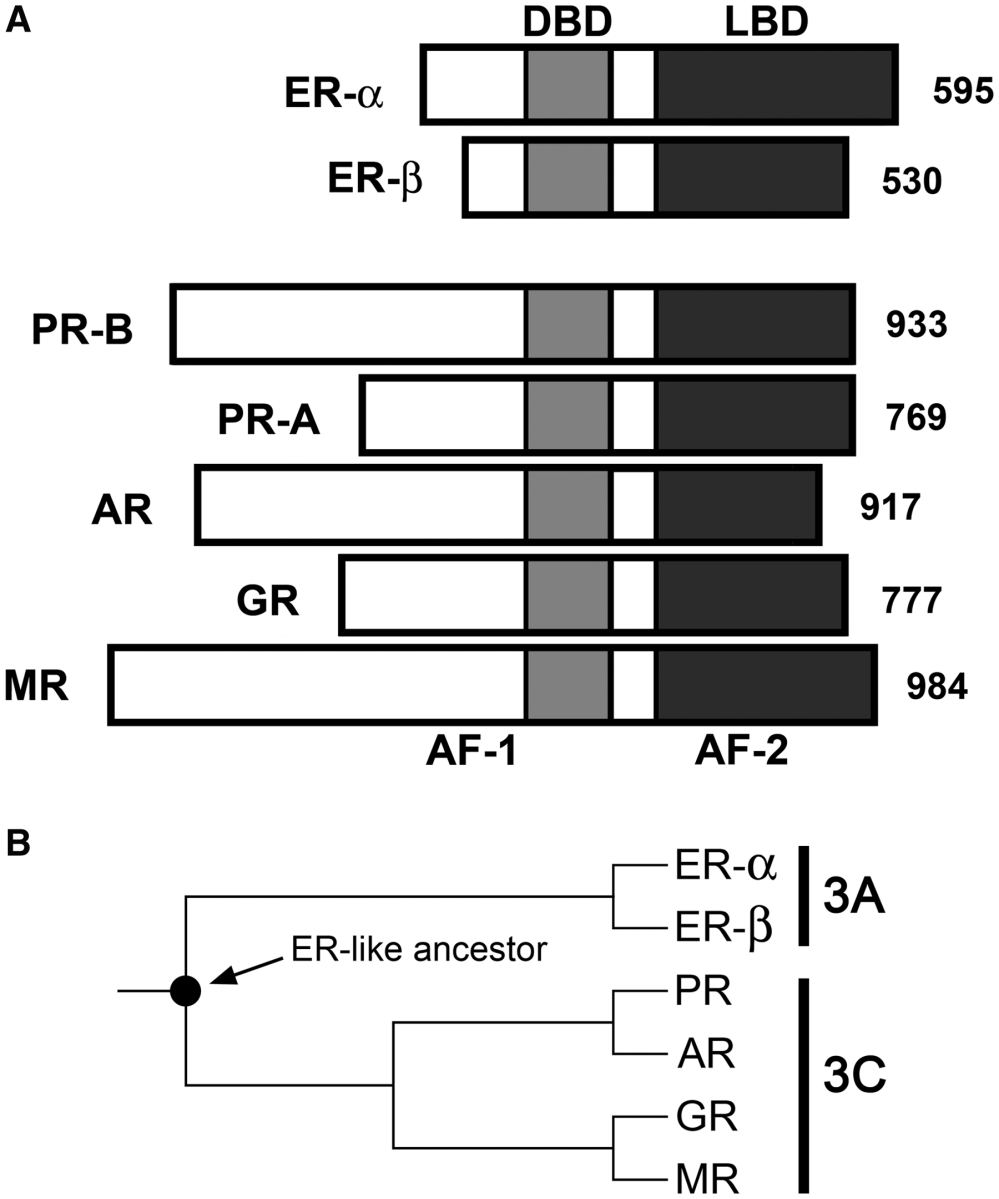
Schematic of steroid receptor domain structure and phylogenetic tree. (**A**) Schematic of steroid receptor domains and number of amino acids. Functional domains as labeled: DBD, DNA binding domain; LBD, ligand-binding domain; AF, activation functions are present in both the *N*-terminal region and LBD. (**B**) Phylogenetic tree representing divergence of the steroid receptor family. Filled circle represents the ER-like common ancestor for subfamily 3A (ER-α and ER-β) and subfamily 3C (PR, AR, GR and MR). The two PR isoforms are not shown, as they are generated from the same gene via alternate transcriptional or translational start sites.

Steroid receptors comprise a family of ligand-activated transcription factors (1). Included are the androgen receptor (AR), estrogen receptor (ER), glucocorticoid receptor (GR), mineralocorticoid receptor (MR) and progesterone receptor (PR). ER exists naturally as two isoforms (ER-α and ER-β) as does PR (PR-A and PR-B). As shown in Figure 1A, all receptors share a common modular structure. Centrally located is a highly conserved DNA binding domain (DBD); C-terminal to the DBD is the ligand-binding domain (LBD). Activation functions are located within the LBD and the *N*-terminal regions. As shown in Figure 1B, phylogenetic studies demonstrate that all receptors descend from a common ER-like ancestor, with AR, GR PR and MR forming subgroup 3C, and the two ER isoforms forming the more distantly related subgroup 3A (2).

The generally accepted framework for receptor function is that on binding ligand, receptors dimerize, bind hormone response elements (HREs) located within upstream promoter sites and recruit coactivating proteins to activate transcription. Although this model provides a strong qualitative understanding, it nonetheless remains incomplete. Specifically lacking is a quantitative perspective for considering receptor-specific gene regulation: how does a homologous family of transcription factors, capable of binding identical or nearly identical response elements, regulate different gene networks? Although we have qualitative insight into aspects of this question, a quantitative understanding—one that yields physical predictions and mechanisms—has yet to be determined.

One reason why such an understanding is lacking is due to the great complexity associated with receptor function. For example, the number of transcription factors that assemble at receptor-regulated promoters likely approaches 50 (3,4); receptor interactions at response elements are highly dynamic both in time and structure (5,6); allosteric coupling and chromatin play significant regulatory roles (5,7); and functional HREs are located not only within promoters but also scattered throughout intronic and intergenic regions (8). Such complexity suggests that a quantitative and bottom-up approach to describing receptor-specific transcriptional properties, similar to that achieved for classical model systems (9), is unlikely to be forthcoming. However, as we will attempt to show, this conclusion may be premature.

Our goal here is to highlight recent advances in placing receptor-specific functional behavior on a more quantitative footing. We focus largely on the role of macromolecular interaction energetics. We first discuss the limitations associated with traditional approaches for assessing the role of binding affinity with functional outcomes such as transcriptional activation. We next demonstrate how rigorous *in vitro* measurements and straightforward interaction models quantitatively relate energetics to transcriptional activity within the cell and discuss why such an approach is unexpectedly effective in describing complex functional behavior. Finally, we examine the implications of these findings for understanding receptor-specific transcriptional regulation.

### Simple binding models predict non-linear affinity-function relationships

**Figure 2.**
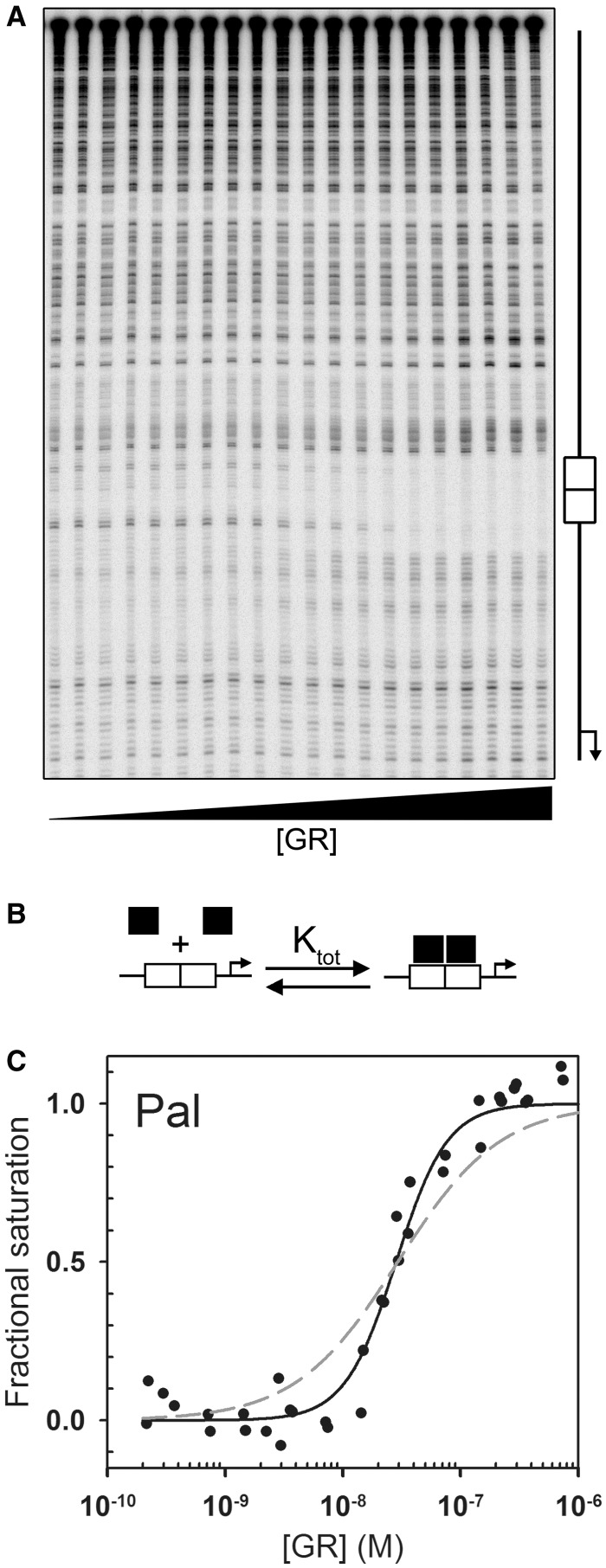
Quantitative analysis of GR-HRE binding energetics. (**A**) Representative quantitative footprint titration image of GR binding to the Pal sequence. Schematic to the right indicates position of the HRE and approximate location of the transcriptional start site. Fractional saturation (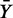) was determined by integrating band intensities across the entire HRE. (**B**) GR-HRE assembly model depicting the total binding reaction and macroscopic product constant (K_tot_), the total affinity for assembling two GR monomers at a palindromic response element. (**C**) Fractional saturation of the Pal sequence from two independent footprint titrations. Solid line represents global fit to both data sets using the K_tot_ binding model in Panel (B) and Equation 2 (SD = 0.087); dashed line represents fit to Equation 1 (SD = 0.126).

**Figure 3.**
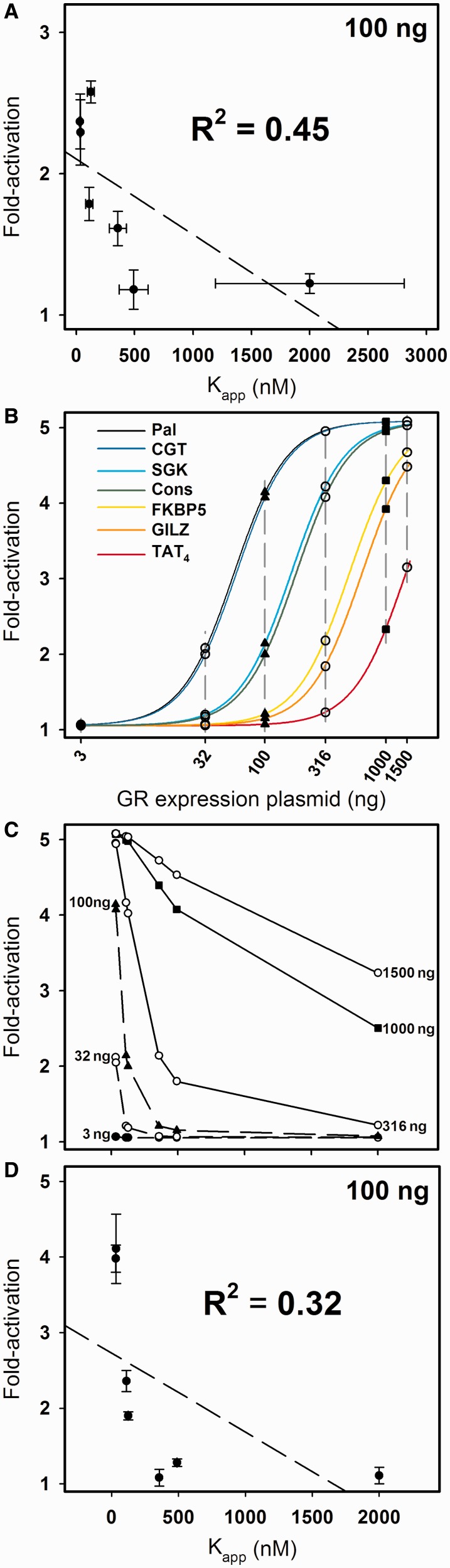
K_app_ versus fold-activation for seven HREs. (**A**) Plot of fold-activity values ± SEM for the seven HRE sequences shown in Table 1; dashed line represents linear regression. An identical R^2^ result is obtained if the data are plotted as a function of total binding affinity (K_tot_). (**B**) Plot of simulated fold-activity for the seven HREs as a function of nanogram GR expression vector. Data points and dashed lines represent cross-sectional analysis used to generate plot in following panel. (**C**) Plot of simulated fold-activities as a function of K_app_ for five GR expression vector doses (3, 32, 100, 316, 1000 and 1500 ng). (**D**) Plot of simulated error-perturbed fold-activities ± SEM (*n* = 3) for the seven HRE sequences at 100 ng GR expression vector dose; dashed line represents linear regression. Error added was identical to that in panel (A); see (10).

**Figure 4.**
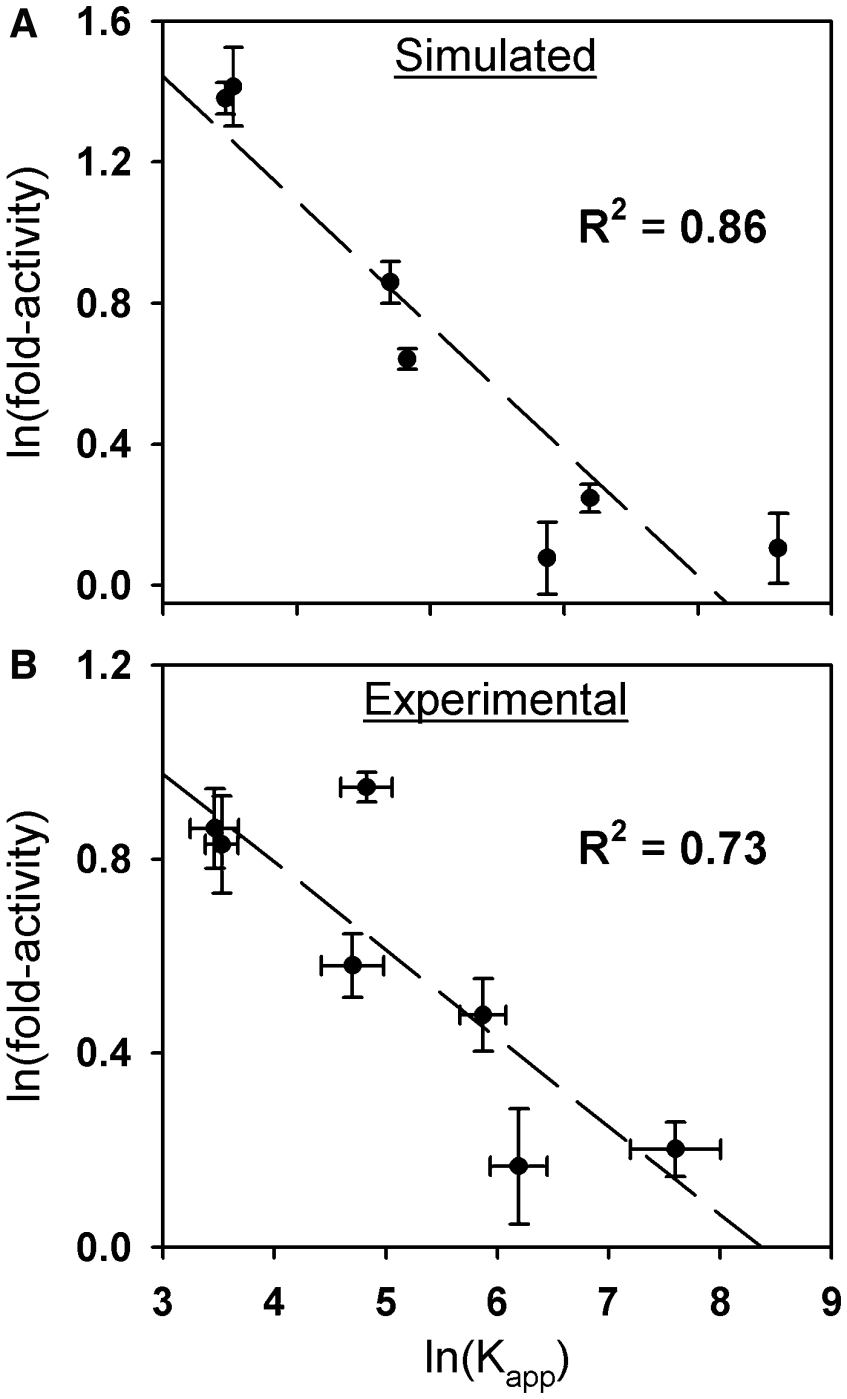
Linear regression of log-transformed fold-activities and K_app_ values at 100 ng GR expression vector. (**A**) Log–log transformation of the experimental data presented in Figure 2A. (**B**) Same as Panel (A) using the simulated error-perturbed data in Figure 2D. For both panels, the same R^2^ value is obtained if the data are analyzed in units of K_tot_ rather than K_app_.

**Figure 5.**
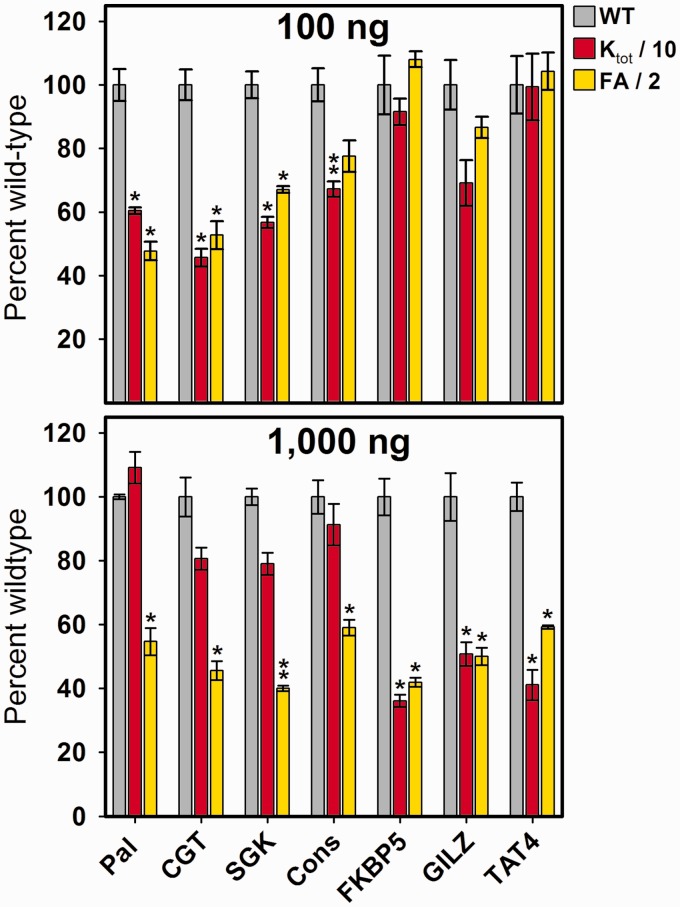
Relative fold-activities for seven HREs as a function of simulated mutagenesis and coactivator knockdown. Simulated activity differences relative to wild-type (gray) for 100 (top) and 1000 (bottom) ng GR-expression vector doses, when K_tot_ is reduced 10-fold (red) or fold-activity (FA) is reduced 2-fold (yellow) (*P < 0.05; **P < 0.005).

**Figure 6.**
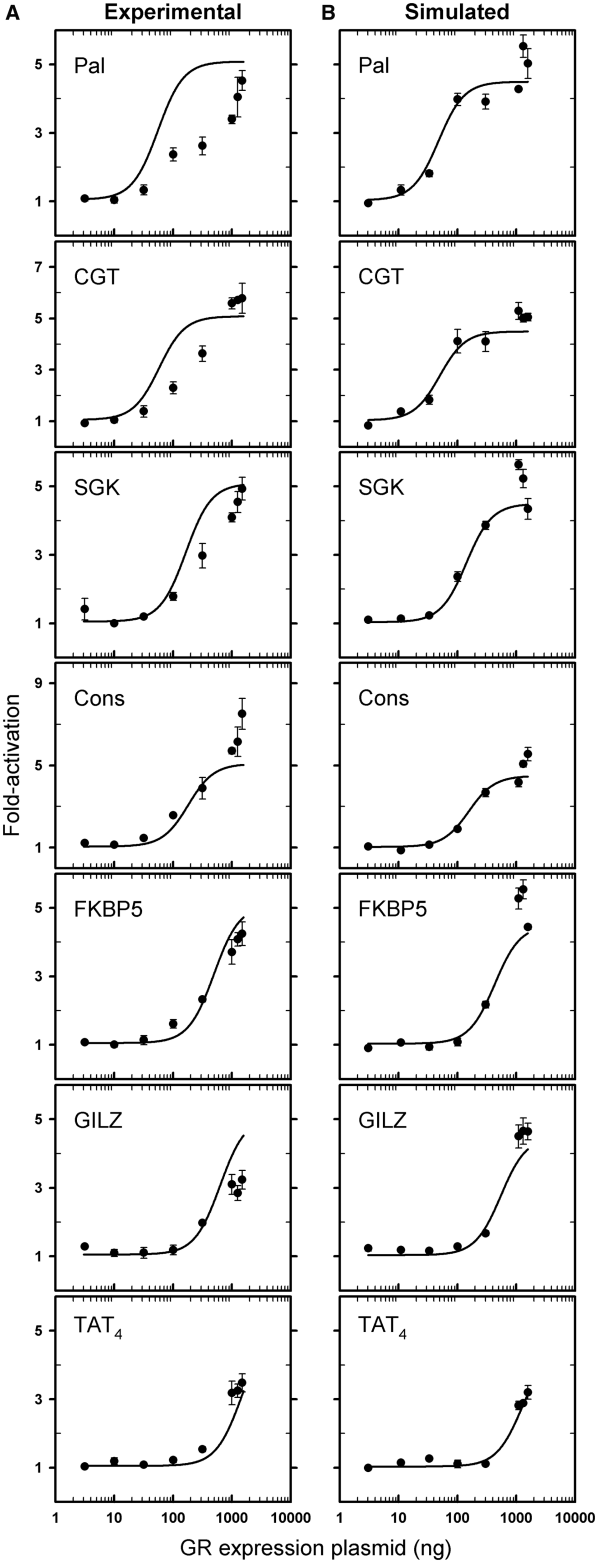
Global fitting of dose–response curves indicates that DNA binding energetics largely account for sequence-specific transcriptional activation. (**A**) Plots of dose–response curves for seven HREs as a function of GR expression vector dose. Filled circles indicate fold-activation ± SEM (*n* = 3). Lines represent global fit of all dose–response curves using a statistical thermodynamic dimer-binding model, K_tot_ for each respective HRE and global scaling factors. (**B**) Same as Panel (A) using simulated error-perturbed fold-activation values. Error was identical to that in panel (A). The data shown here were also used to determine the extent of correlation presented in Table 2.

**Figure 7.**
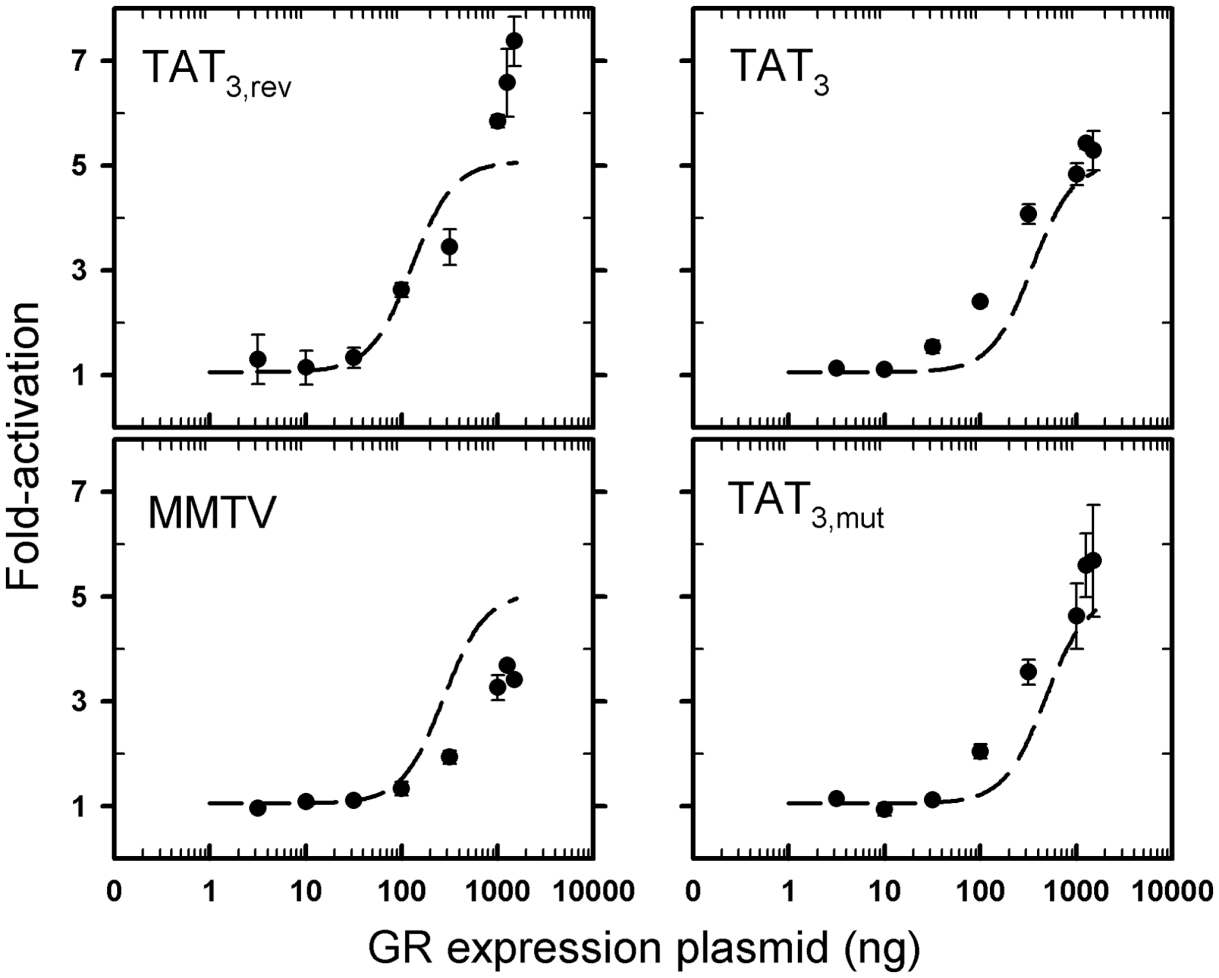
Predicted and experimentally determined dose–response curves for four additional HREs. Dose–response curves ± SEM (*n* = 3) for GR-induced activity for the four HREs (filled circles). Dashed lines represent predicted dose–response curves using respective K_tot_ determined previously (10) and *d*, *e* and *f* scaling factors resolved in Figure 6A. DNA sequence and experimentally determined GR binding affinity for the four HREs were previously presented (10).

**Figure 8.**
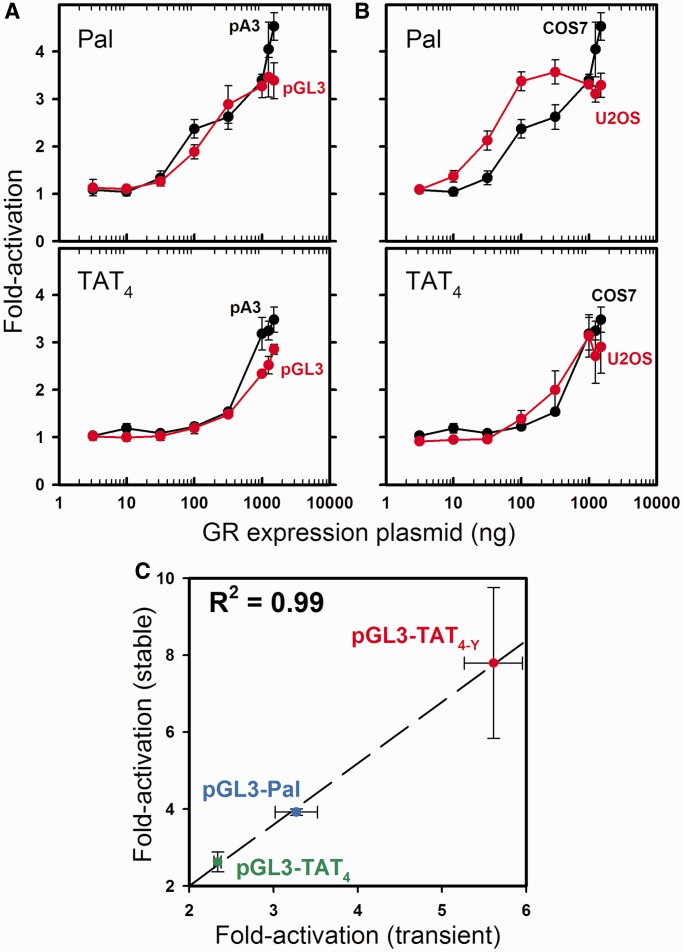
Sequence-specific activation is maintained in multiple promoter-types, cell lines and in chromatin environment. (**A**) pA3-Pal and pA3-TAT_4_ dose–response curves in COS7 cells (black) overlaid with dose–response curves ± SEM (*n* = 3) from the respective sequences in pGL3 vector (red). (**B**) pA3-Pal and pA3-TAT_4_ dose–response curves in COS7 cells (black) overlaid with dose–response curves ± SEM (*n* = 3) for respective sequences in U2OS cells (red). (**C**) TA-induced activity ± SEM (*n* ≥ 2) of pGL3-TAT_4_ (green), pGL3-Pal (blue) and pGL3-TAT_4-Y_ [red; (10)] determined in transient and stably transfected COS7 cells (1 μg GR expression vector). Dashed line represents linear regression.

Steroid receptors recognize an array of HREs, typically imperfect palindromes that vary by one or more base pairs. To explore the relationship between receptor–DNA interaction energetics and transcriptional regulation, we determined the energetics of GR binding to seven previously characterized HREs (10,11). Shown in Figure 2A [Figures 2–8 and Tables 1–2 reproduced with permission (10)] is a representative titration of one such sequence (Pal), determined using quantitative footprint titration (12,13). Two models were used to fit the data. The first is the Langmuir binding model, which resolves the apparent binding affinity (K_app_):
(1)
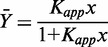

where 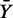 is the fractional saturation at the response element, and *x* is the free receptor monomer concentration. The second model, schematically depicted in Figure 2B, resolves K_tot_, the total affinity for assembling two GR monomers at the palindromic HRE:
(2)
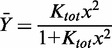

where *x* is again in units of free monomer concentration.

**Table 1.** HRE sequences, apparent and total GR binding affinities and ligand-induced fold-activation at 100 ng dose of GR expression vector

HRE	Sequence	K_app_ (nM)^a^	K_tot_ (fM^2^)^a^	Fold-activation^b^
Pal	AGAACAaaaTGTTCT	32 ± 7	0.800 ± 0.134	2.4 ± 0.19
CGT	AGAACAtttTGTACG	34 ± 5	0.877 ± 0.123	2.3 ± 0.23
SGK	AGAACAtttTGTCCG	110 ± 31	7.09 ± 1.75	1.7 ± 0.12
Cons	AGAACAaaaTGTACC	125 ± 29	8.55 ± 1.42	2.6 ± 0.08
FKBP5	AGAACAgggTGTTCT	356 ± 73	64.9 ± 7.80	1.6 ± 0.12
GILZ	AGAACAttgGGTTCC	490 ± 125	105 ± 16.4	1.2 ± 0.14
TAT_4_	AGAACAtcccTGTACA	2,000 ± 809	558 ± 10.4	1.2 ± 0.07

^a^Apparent (K_app_) and total (K_tot_) GR-HRE binding affinities ± standard deviation (SD).

^b^Fold-activation ± standard error of the mean (SEM).

**Table 2.** Log-transformed correlation analysis of K_app_ and fold-activation for seven HRE sequences^a^

	Experimental		Simulated	
DNA (ng)	R^2^	P	R^2^	P
3	0.01	0.80	0.14	0.40
10	0.34	0.17	0.12	0.44
32	0.63	0.03	0.44	0.10
100	0.73	0.01	0.86	≤0.01
316	0.66	0.03	0.90	≤0.01
1000	0.31	0.19	0.37	0.14
1259	0.44	0.11	0.56	0.05
1500	0.40	0.10	0.61	0.04

^a^R^2^—coefficient of determination; *P*—probability that R^2^ results from two uncorrelated variables.

The experimental isotherm generated from the footprint titration and best-fit binding curve to both models are shown in Figure 2C. The total and apparent binding affinities for Pal and the six remaining response elements are summarized in Table 1. It is clear that the Langmuir model poorly describes the data. The reason is that apparent affinity does not take into account GR dimerization at the palindrome (14). Consequently, although the K_app_ for each sequence suggests that the range of GR binding affinities spans ∼60-fold, the K_tot_ values demonstrate the true range is nearly 700-fold.

To assess the relationship between *in vitro* DNA binding energetics and transcriptional activity in the cell, we next measured the ligand-dependent increase in transcriptional activity (‘fold-activation’) for each response element by transient transfection (see Table 1). Using the traditional approach for assessing the role of binding affinity in receptor function (11,15), we plotted the seven GR affinities versus the level of fold-activity induced by each sequence. As seen visually in Figure 3A and as indicated by linear correlation analysis, it appears that there is only a weak relationship (R^2 ^= 0.45). A similar lack of correlation has been reported previously for AR, the ER isoforms and GR (11,15,16). A second observation is that sequences with statistically identical affinities toward the receptor (e.g. SGK and Cons) nonetheless generate statistically different functional responses. This phenomenon was first observed for ER-α (17), being consistent with the then emerging hypothesis that individual response elements act as unique allosteric effectors (18).

These results appear to show that DNA binding affinity makes only a small contribution to transcriptional activity. However, the experiments in Figure 3A do not necessarily reflect an accurate comparison: the transfection assay measures activity at only a single GR expression dose, whereas the DNA binding measurements reflect a wide range of GR concentrations. Therefore, to more rigorously examine this relationship, we generated simulated dose–response curves for the seven response elements analyzed earlier. We used a simple model in which the energetics of receptor binding to each response element generate a maximal 5-fold increase in transcriptional activity (10). As shown in Figure 3B, these curves are identical in shape to a binding curve but are in units of mass receptor expression vector and fold-transcriptional activity.

Transformation of the curves into a traditional affinity-function plot simply requires choosing a particular dose of receptor expression vector, identifying the fold-activity associated with that dose and plotting it against the receptor–DNA binding affinity associated with each response element. For the simulated dose–response curves, six doses are represented as dashed vertical lines, all forming cross-sections through the curves. As seen in Figure 3C, the resultant plots for the six doses are, not surprisingly, highly dependent on where the particular cross-section is taken. More unexpectedly, the relationship between affinity and function is never linear as is implicitly assumed in the traditional correlation analysis, but is instead highly non-linear. In fact, expression vector doses most sensitive to changes in affinity, and thus most useful for analysis (e.g. 100 ng), generate the most extreme non-linearity. Moreover, at such doses, the exponential-like decay in activity provides an appealing explanation for how response elements with similar affinities can generate different functional activities. Non-linearity is not a result of the model used to generate the data but is a general consequence of transforming any hyperbolic or sigmoidal-shaped dose–response curve.

We next asked how experimental error influences these results. Using the identical type of error found in the experimental data shown in Figure 3A, we error-perturbed the simulated data (10). The results for the 100 ng dose are plotted in Figure 3D. The error-perturbed simulated data are strikingly similar to the experimental data, again showing only a weak correlation between affinity and function (R^2 ^= 0.32). Moreover, response elements with similar receptor-binding affinities again generate statistically different functional activities. Thus, the simulations demonstrate that linear statistical tests are inappropriate for assessing affinity-function relationships. More intriguingly, they suggest that experimental results as shown in Figure 3A do not necessarily arise from complex mechanisms.

One commonly used approach for assessing correlation in the case of non-linear relationships is to simply linearize the data via log-transformation (19,20). If this is applied to the simulated data in Figure 3D, the correlation between DNA binding affinity and transcriptional activity is now accurately seen as being strong and statistically significant (R^2 ^= 0.86 and *P* ≤ 0.01; Figure 4A). A similar result is found for the experimental data (R^2 ^= 0.73 and *P* = 0.01; Figure 4B). Thus, contrary to initial impressions, GR-DNA binding affinity and transcriptional activity are highly correlated. Noting that the DBD of all steroid hormone receptors is highly conserved and that the receptors bind identical or nearly identical response elements, we anticipate that similar results will be found with the remaining receptors.

Unfortunately, although log-transform and other types of linearization approaches (10,19) correctly reveal the correlation in this specific instance, they are still of limited utility. This is due to their well-known distorting effect on quantitative relationships (14). For example, as shown in Table 2, log-transformation of simulated error-perturbed data—generated using a model in which DNA binding affinity fully governs transcriptional activity—indicates that at most doses there is still no statistical significance between affinity and function. The reason for this is simply that most high or low GR expression doses are insensitive to changes in affinity and function compared with intermediate doses. This is so because cross-sections taken at or near the plateaus of fold-activity offer little correlative insight. Thus, the ability of log-transformation to discern a statistically significant correlation is constrained within a very small range; we find only a threefold range in which linearization is a legitimate analytical method (10). Yet, to determine which doses are amenable to such an approach, it is first necessary to generate complete dose–response curves for all DNA sequences of interest, and then empirically identify that small range sensitive to the affinity-function relationship. Because this extent of study is rarely performed, traditional analyses as carried out in Figure 3A or linearization analyses using the “correct” log-transformation approach in Figure 4 are quite likely to generate incorrect conclusions.

### Non-linearity and implications for other experimental approaches

The non-linear relationship between DNA binding affinity and transcriptional output has a number of interesting consequences for other assays used to probe receptor behavior. As an example, for the seven HREs in Table 1, we simulated dose-response data in which GR binding affinity (K_tot_) was uniformly reduced by 10-fold for all sequences. This might occur via mutational analysis of either the receptor or the DNA. We also simulated data in which the maximal fold-activity associated with all HREs was uniformly reduced 2-fold, as might result from a coactivator-knockdown experiment. For two GR expression doses (100 and 1000 ng), the resultant fold-activities for each response element are shown normalized to wild-type (Figure 5).

Even though the changes in either affinity or activity were applied identically to all response elements, the data at 100 ng appear to indicate that only a subset is affected. A different subset is affected at 1000 ng, but only by the change in binding affinity—the change in fold-activity by coactivator knockdown is now accurately seen as similar for all sequences. Thus, different doses of GR expression vector generate variable results. This occurs because a low dose of GR expression vector preferentially reveals functional changes only at high-affinity binding sites and vice versa. In conclusion, if receptor-mediated transcriptional activity is under energetic control (a hypothesis we rigorously test in the next section), then seemingly independent functional assays may generate equally problematic results. This is because the assays share the common attribute of examining transcriptional activity at a single expression dose.

### Equilibrium models readily describe complex cellular function

A more powerful alternative, that bypasses the limitations of single-dose approaches, is to directly fit multiple sets of functional data over a wide range of receptor concentrations using molecular-based interaction models. We illustrate this approach using the example of GR and its interactions with multiple HREs. We first experimentally determined complete dose–response curves for the seven response elements shown in Table 1. We then globally fit the curves to a simple equilibrium binding model: the experimentally determined GR binding affinity (*K_tot,i_*) for each response element *i* was a fixed parameter, but the maximal (*d*) and minimal (*e*) fold-activities were allowed to float to values common for all response elements:
(3)
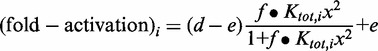



Equation 3 is similar to that used to fit the DNA binding data, but the overall expression is modified by scaling factors *d, e* and *f*. These parameters allow the iosotherms determined from the *in vitro* footprint titration experiments to be numerically fit to their respective *in situ* fold-activation curves. Thus, the isotherms are treated as transition curves, where *d* rescales the *y*-axis amplitude taking into account the actions of all other transcription factors, *e* shifts the *y*-axis baseline, and *f* converts the *x*-axis from GR concentration in molar units to GR expression vector in nanogram units. Because *d, e* and *f* are global parameters common to all data sets, all binding curves are rescaled identically.

Shown in Figure 6A are the experimental dose–response curves associated with each of the seven response elements (filled circles) and the best-fit curves for the respective sequences as determined from the global analysis (solid lines). For comparative purposes, we also used the same fitting model, DNA binding energetics, extent of error and resolved maximal and minimal fold-activation values to generate simulated dose–response curves. We then fit these curves using the approach applied to the experimental data. We emphasize that these results, presented in Figure 6B, represent the expected outcome if GR-DNA binding energetics are the exclusive contributor to sequence-specific transcriptional activity. The simulations, thus, serve to critically evaluate the forces underlying the transcriptional data.

By visual inspection, the DNA binding model describes well almost all of the cellular data. This is despite the fact that all HREs are assumed to generate identical maximal and minimal fold-activities, and that the true range of DNA binding affinities spans nearly 700-fold. Moreover, we note that the data and fit are similar to the simulations of Figure 6B. However, to determine whether these findings were unique only to these sequences, we examined four additional sequences—an HRE placed in reverse orientation (TAT_3,rev_), two naturally occurring HREs (MMTV and TAT_3_) and a synthetic mutated HRE (TAT_3,mut_). Using the best-fit maximal and minimal fold-activities in Figure 6A, the same binding model and the experimentally measured DNA binding affinities for the four response elements, we generated predicted dose–response curves (dashed lines). As shown in Figure 7, the predictions capture the overall trends of the data. Thus, a simple equilibrium model that assumes that DNA binding energetics dictate sequence-specific transcriptional output is sufficient to describe the functional behavior of nearly a dozen response elements.

To determine whether these results were a fortuitous consequence of our experimental conditions, we used the highest and lowest affinity response elements from Figure 6 (Pal and TAT_4_) to measure GR transcriptional activity in a different promoter context, cell line and chromatin environment. Shown in Figure 8A are the dose–response curves for Pal and TAT_4_ carried out in COS7 cells using a pA3 promoter (black; derived from a minimal thymidine kinase promoter sequence). Overlaid are analogous measurements generated using a pGL3 promoter (red; derived from a minimal SV40 promoter sequence). Only subtle differences are seen, suggesting that promoter context has little influence on sequence-specific transcriptional activity. Shown in Figure 8B are again the pA3 dose–response curves for Pal and TAT_4_ in COS7 cells (black); now overlaid are the analogous measurements in U2OS cells (red). Although slight differences are observed for the Pal sequence, the trend of Pal being a stronger activator than TAT_4_ is maintained. Finally, in Figure 8C we tested the influence of chromatin by stably transfecting three HRE constructs into COS7 cells. Transcriptional activity of the pooled cell population was then compared with that measured by transient transfection; we see excellent correlation (R^2 ^= 0.99).

### Why are equilibrium measurements unexpectedly effective at describing complex behavior?

Collectively, the results presented in Figures 6–8 demonstrate that affinity-based gene control is a general feature of GR function. This would seem to be unexpected noting the significant complexity associated with receptor-mediated transcriptional activity. How can a simple equilibrium-binding model describe complex cellular behavior? As will be described elsewhere using a more quantitative and theoretical approach, the ability of energetics to describe GR activity indicates that such activity must be under thermodynamic control. That is, of the many interactions that link transcription factor-promoter assembly with gene output, the rate of receptor binding to the DNA must be fast relative to the loading rates of other factors. Phillips and coworkers elegantly describe this as a ‘separation of timescales’ (21). If such a prediction holds for GR and other steroid receptors, this implies that receptor–promoter occupancy is dictated by the *in situ* equilibrium concentrations of receptor and promoter. This will be so even if the active receptor concentration is fluctuating owing to translation, post-translational modification or degradation events. That is, based on the timescale argument, receptor–promoter equilibration rates must be faster than the rates at which receptor concentrations are changing—once more, a quantitatively testable prediction.

### Energetics and receptor-specific gene regulation

Noting the strong role of energetics in GR function and that the remaining steroid receptors comprise a phylogenetically related family, we have speculated that differences in promoter-binding energetics among family members could play a role in receptor-specific function (22,23). In particular, might such differences account for the paradoxical ability of receptors to bind largely identical response elements yet regulate different subsets of genes (24–26)? To examine this possibility, we are systemically dissecting the promoter-binding energetics of all the human steroid receptors, at a ‘standard state’ condition under which the receptors are amenable to rigorous and comparative analysis (22,27–30).

**Figure 9.**
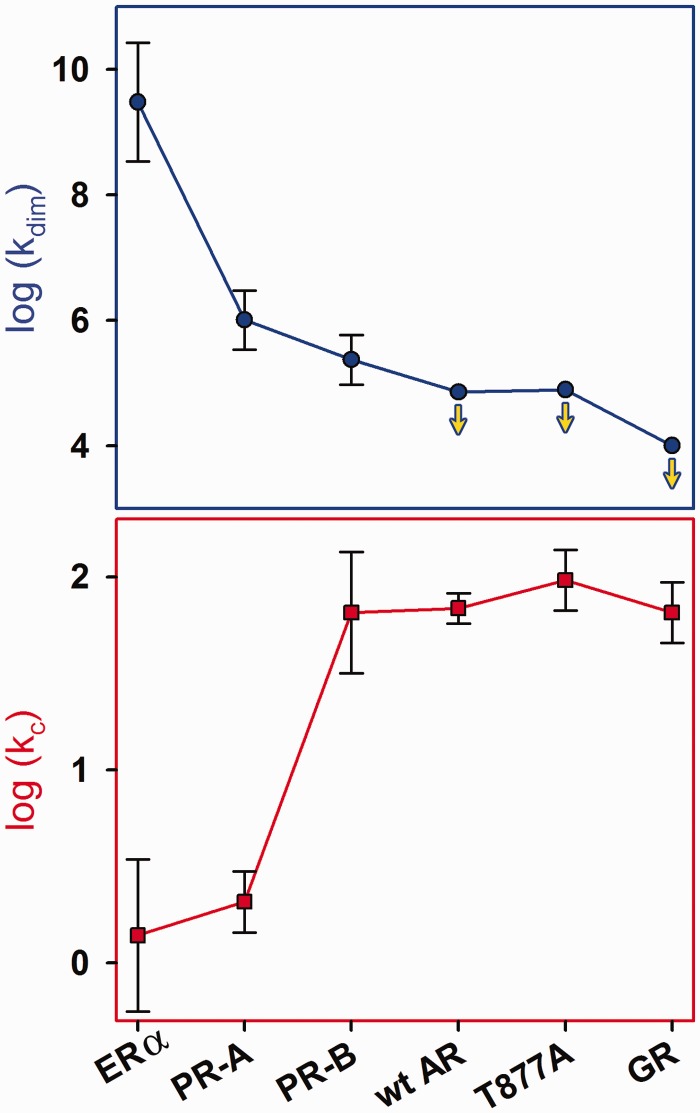
Microstate energetics of steroid receptor assembly at a simple two-site promoter. Circles represent receptor dimerization affinity (k_dim_) and squares represent inter-site cooperativity (k_c_). As dimerization was not observed for wild-type AR, T877A and GR, downward arrows have been added to indicate that plotted values represent lower limits. Error bars represent 67% confidence intervals. Because the dimerization and cooperativity terms each represent a microscopic rather than a macroscopic interaction (e.g. K_tot_), they are represented by a lower case *k*.

By the traditional functional model (1), receptors dimerize in the absence of DNA (k_dim_) and bind to response elements as pre-formed dimers. Assembly at a multisite promoter may also be coupled to inter-site cooperativity (k_c_). For full-length human ER-α and the two PR isoforms, these values were determined using a simple two-site promoter and under our standard state conditions (pH 8.0, 100 mM NaCl and 4°C); the results are plotted in Figure 9. Also shown are the cooperativity terms resolved for full-length AR, an AR point mutant associated with advanced prostate cancer (T877A) and GR. Interestingly, these receptors did not show any evidence for dimerization, allowing us to only plot lower limits for assembly affinity.

Despite being a homologous family of transcription factors, the results make clear that under identical conditions, receptor dimerization and cooperative energetics vary enormously. Moreover, dimerization energetics are generally inversely proportional to cooperativity. For example, ER-α displays a sub-nanomolar dimerization affinity but generates non-existent cooperativity. By contrast, the AR proteins and GR have dimerization limits that are at least four to five orders of magnitude weaker than ER-α, and cooperativity values two orders of magnitude greater. Of further interest, the inverse relationship between dimerization and cooperativity trends along evolutionary lines. Thus PR-B, AR, T887A and GR—all closely related subgroup 3C receptors (see Figure 1B)—partition their dimerization and cooperative energetics similarly. In sharp contrast, ER-α, a subgroup 3A receptor, maintains a distinctly different distribution. (It is also clear that PR-A behavior is not fully consistent with this argument, as it exhibits weak cooperativity. We note that PR-A is still capable of generating 1000-fold cooperative stabilization on a different promoter architecture (28,31)*.* We find similar promoter-specific results for the remaining subgroup 3C receptors. By contrast, ER-α cooperativity is not detectable on seven promoter architectures we have tested to date).

**Figure 10.**
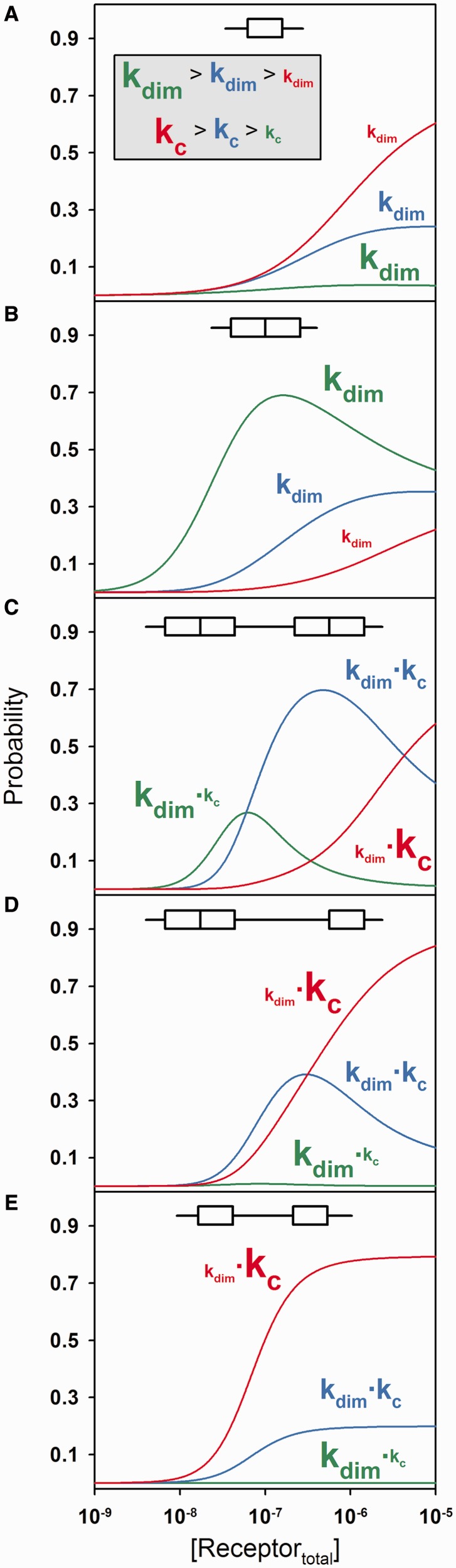
Predicted probabilities of the fully ligated promoter state under competitive binding conditions. (**A**) Simulation of competitive binding to an isolated half-site by three receptors differing in dimerization and cooperative energetics. Red, k_dim_ = 10 μM and k_c_ = 200; Blue, k_dim_ = 1 μM and k_c_ = 50; Green, k_dim_ = 16 nM and k_c_ = 1. Affinity of monomer binding to half-site was assumed to be an identical 1 μM for all receptors. Strength of dimerization and cooperativity terms is indicated schematically by font size of each parameter. (**B**) Same as (A) but now binding to an isolated palindrome. Affinity of pre-formed dimer binding was assumed to be an identical 10 nM for all receptors. (**C**) Same as above, but binding to a promoter containing two palindromic sites. (**D**) Same as above, but binding to a promoter containing one half-site and one palindrome. (**E**) Same as above, but binding to a promoter containing two half-sites.

These results suggest that differences in promoter-binding energetics are an evolutionarily conserved feature of the receptor family, and thus critical to function. They also suggest a basis for receptor-specific promoter function via differential promoter occupancy. To illustrate, we simulated the probability of receptor assembly at several different promoter architectures under conditions in which multiple receptor populations are competing for identical sites. For example, at an isolated half-site (Figure 10A), receptors with weak or non-existent dimerization energetics such as AR or GR easily outcompete receptors with stronger dimerization energetics; the reverse is true for an isolated palindrome (Figure 10B). For multisite promoters, preferential binding can only be achieved by allowing differences in both dimerization energetics and cooperativity, entirely consistent with the receptor-specific differences we observe experimentally. For example, a promoter containing multiple palindromic binding sites allows for preferential occupancy of a single receptor (blue) via moderate contributions from both parameters (Figure 10C). However, a promoter containing a half-site and a palindrome results in simultaneous occupancy, and presumably some level of joint gene regulation (Figure 10D). Finally, a promoter containing multiple half-sites allows preferential occupancy of a third receptor (red; Figure 10E).

## CONCLUSIONS

The simulations in Figure 10 indicate that combining different interaction energetics with different promoter architectures generates preferential occupancy by each receptor—even when multiple receptors are competing for identical binding sites. With our findings that homologous receptors partition their energetics in parallel with their phylogenetic divergence, and that DNA binding energetics are the primary contributor to sequence-specific transcriptional activity, nature may have selected for energetic differences as the basis for receptor-specific transcriptional regulation. This implies that steroid receptors are energetically poised to carry out function and predicts a ‘binding affinity landscape’ for each receptor (32). Genome-wide studies of receptor cistromes and transcriptomes are consistent with this interpretation, indicating that monomeric receptors such as AR preferentially bind and activate at half-sites, whereas strongly dimeric receptors such as ER-α are active at palindromes (8). Furthermore, natural promoters seem to invariably contain multiple receptor binding sites, implying a role for cooperative protein–protein interactions. Future challenges center on the thermodynamic and kinetic coupling between receptor–DNA assembly, coactivator recruitment and amplitude of transcriptional activation. In this regard, more sophisticated statistical thermodynamic models will be necessary. However, this does not imply that every interaction associated with transcriptional output needs to be accounted for—in the case of steroid receptor-specific gene regulation, receptor–DNA energetics appear to be paramount.

## FUNDING

National Institutes of Health (NIH) [DK88843 to D.L.B.]. Funding for open access charge: NIH [DK88843 to D.L.B.].

*Conflict of interest statement*. None declared.
